# A new synergistic relationship between xylan-active LPMO and xylobiohydrolase to tackle recalcitrant xylan

**DOI:** 10.1186/s13068-020-01777-x

**Published:** 2020-08-10

**Authors:** Anastasia Zerva, Christina Pentari, Sacha Grisel, Jean-Guy Berrin, Evangelos Topakas

**Affiliations:** 1 Industrial Biotechnology and Biocatalysis Group, School of Chemical Engineering, National Technical University of Athens, 9 Iroon Polytechniou Str., Zografou Campus, 15780 Athens, Greece; 2 INRAE, Aix Marseille University, Biodiversité Et Biotechnologie Fongiques (BBF), UMR1163, 13009 Marseille, France

**Keywords:** Synergism, AA14, GH30, Xylanase, Xylobiohydrolase, Lignocellulose

## Abstract

**Background:**

Hemicellulose accounts for a significant part of plant biomass, and still poses a barrier to the efficient saccharification of lignocellulose. The recalcitrant part of hemicellulose is a serious impediment to the action of cellulases, despite the use of xylanases in the cellulolytic cocktail mixtures. However, the complexity and variety of hemicelluloses in different plant materials require the use of highly specific enzymes for a complete breakdown. Over the last few years, new fungal enzymes with novel activities on hemicelluloses have emerged. In the present study, we explored the synergistic relationships of the xylan-active AA14 lytic polysaccharide monooxygenase (LPMO), *Pc*AA14B, with the recently discovered glucuronoxylan-specific xylanase *Tt*Xyn30A, of the (sub)family GH30_7, displaying xylobiohydrolase activity, and with commercial cellobiohydrolases, on pretreated natural lignocellulosic substrates.

**Results:**

*Pc*AA14B and *Tt*Xyn30A showed a strong synergistic interaction on the degradation of the recalcitrant part of xylan. *Pc*AA14B was able to increase the release of xylobiose from *Tt*Xyn30A, showing a degree of synergism (DS) of 3.8 on birchwood cellulosic fibers, and up to 5.7 on pretreated beechwood substrates. The increase in activity was dose- and time- dependent. A screening study on beechwood materials pretreated with different methods showed that the effect of the *Pc*AA14B–*Tt*Xyn30A synergism was more prominent on substrates with low hemicellulose content, indicating that *Pc*AA14B is mainly active on the recalcitrant part of xylan, which is in close proximity to the underlying cellulose fibers. Simultaneous addition of both enzymes resulted in higher DS than sequential addition. Moreover, *Pc*AA14B was found to enhance cellobiose release from cellobiohydrolases during hydrolysis of pretreated lignocellulosic substrates, as well as microcrystalline cellulose.

**Conclusions:**

The results of the present study revealed a new synergistic relationship not only among two recently discovered xylan-active enzymes, the LPMO *Pc*AA14B, and the GH30_7 glucuronoxylan-active xylobiohydrolase *Tt*Xyn30A, but also among *Pc*AA14B and cellobiohydrolases. We hypothesize that *Pc*AA14B creates free ends in the xylan polymer, which can be used as targets for the action of *Tt*Xyn30A. The results are of special importance for the design of next-generation enzymatic cocktails, able to efficiently remove hemicelluloses, allowing complete saccharification of cellulose in plant biomass.

## Background

Recently, accumulating data on the discovery of novel enzyme activities tend to revolutionize the way microbial lignocellulose degradation was perceived during the last 30 years. Major breakthroughs in this field, such as the discovery of lytic polysaccharide monooxygenases (LPMOs, [1]) and more recently enzymes with xylobiohydrolase activity [2, 3] pave the way for major changes in the established theories regarding lignocellulose degradation. More specifically, for hemicellulose, the discovery of xylan-active AA14 LPMOs [4], as well as the conformations of xylan polymers interacting with cellulose [5–7], shed new light on the hemicelluloses structure and degradation pathways. According to a recent study, lytic xylan oxidases belonging to AA14 family selectively oxidize xylan polymers that cover cellulose fibrils, while no activity is detected on soluble xylan polymers, or underlying cellulose fibrils [4]. The explanation for this preference was based on recent discoveries regarding the conformations xylan polymers can receive when in contact with cellulose [6]. The authors hypothesized that AA14 LPMOs are active only on the twofold screw xylan polymers, acting as a shield of cellulose against microbial attack, in contrast with threefold screw soluble xylan polymers. The flat twofold screw conformation of xylan is recalcitrant to degradation by xylanase and displays a certain degree of ‘crystallinity’, which might be necessary for LPMO activity. This hypothesis is in line with earlier reports on the activity of cellulose-acting AA9 LPMOs on cellulose-associated xylan [8]. At the same time, Couturier et al. found that AA14 LPMOs produce only traces of C1-oxidized products, and therefore, the AA14 LPMO activity is detected only in synergism with a GH11 xylanase [4]. AA14 LPMOs also significantly boost the hydrolytic effect of cellulolytic cocktails of standard composition, on woody substrates.

Aside from AA14 LPMOs, which selectively oxidize recalcitrant xylan, AA9 LPMOs have been reported to target xylan associated to cellulose [8, 9]. A single LPMO belonging to AA9 family has been reported to oxidize isolated xylans (PMO9A_MALCI, [10]), together with a bacterial AA10 LPMO (*Kp*LPMO10A, [11]), although with different regioselectivity; *Kp*LPMO10A released exclusively C4-oxidized sugars, while PMO9A_MALCI was shown to produce both C1- and C4-oxidized products.

On the other hand, the recent discovery of the first fungal enzymes with xylobiohydrolase activity [2, 3] opens up new pathways for xylan degradation. GH30_7 xylanases not only act specifically on glucuronoxylan in an endo-manner, releasing uronic acid-decorated xylo-oligosaccharides (UXOS), but they can also act in an exo-manner, releasing xylobiose [2, 3] from the non-reducing end, or xylose from the reducing end [12] of xylan polymers, or xylo-oligosaccharides (XOS). The recent discovery of such novel enzymatic activities raised numerous questions regarding the role and significance of such xylobiohydrolases in the decomposition of lignocellulose in natural ecosystems, and also regarding the potential of such enzymes in the design of biomass-degrading enzyme mixtures. Moreover, the activity of GH30_7 xylanases in natural woody substrates could shed light on the substrate preference and biological role of such novel enzymes.

Aside from the obvious significance of experimental data on single enzymes, the recalcitrant and complex structure of plant cell walls requires the coordinate action of a broad range of enzymes with varied specificities, acting synergistically, for the complete degradation of biomass. Therefore, the focus on enzyme combinations and their effects on lignocellulose is of equal importance. Enzyme synergism is the enhancement of substrate conversion observed through the combined action of two or more enzymes. Some enzyme combinations with strong synergistic effects are well established in the literature, such as the cellulases–xylanases synergism and the synergism among the cellulose-acting enzymes, such as CBHs and EGs [13, 14]. Thus, it is widely accepted that xylan-degrading enzymes are indispensable in LCB degradation, because the removal of xylan increases the accessibility of cellulose-binding sites for cellulases [15, 16]. On the other hand, the synergism between cellulose-acting LPMOs and cellulases is well documented [13, 17, 18].

In the present work, the role of a xylan-active LPMO from *Pycnoporus coccineus* (*Pc*AA14B) in the degradation of (hemi)cellulose was investigated through exploration of its synergistic relationships with a xylanase of the GH30_7 family (*Tt*Xyn30A) from *Thermothelomyces thermophila* (previously named *Myceliophthora thermophila* and *Sporotrichum thermophile*), and commercial cellobiohydrolases, on natural lignocellulosic substrates.

## Results

### Synergism between *Pc*AA14B LPMO and *Tt*Xyn30A xylanase

**Fig. 1:**
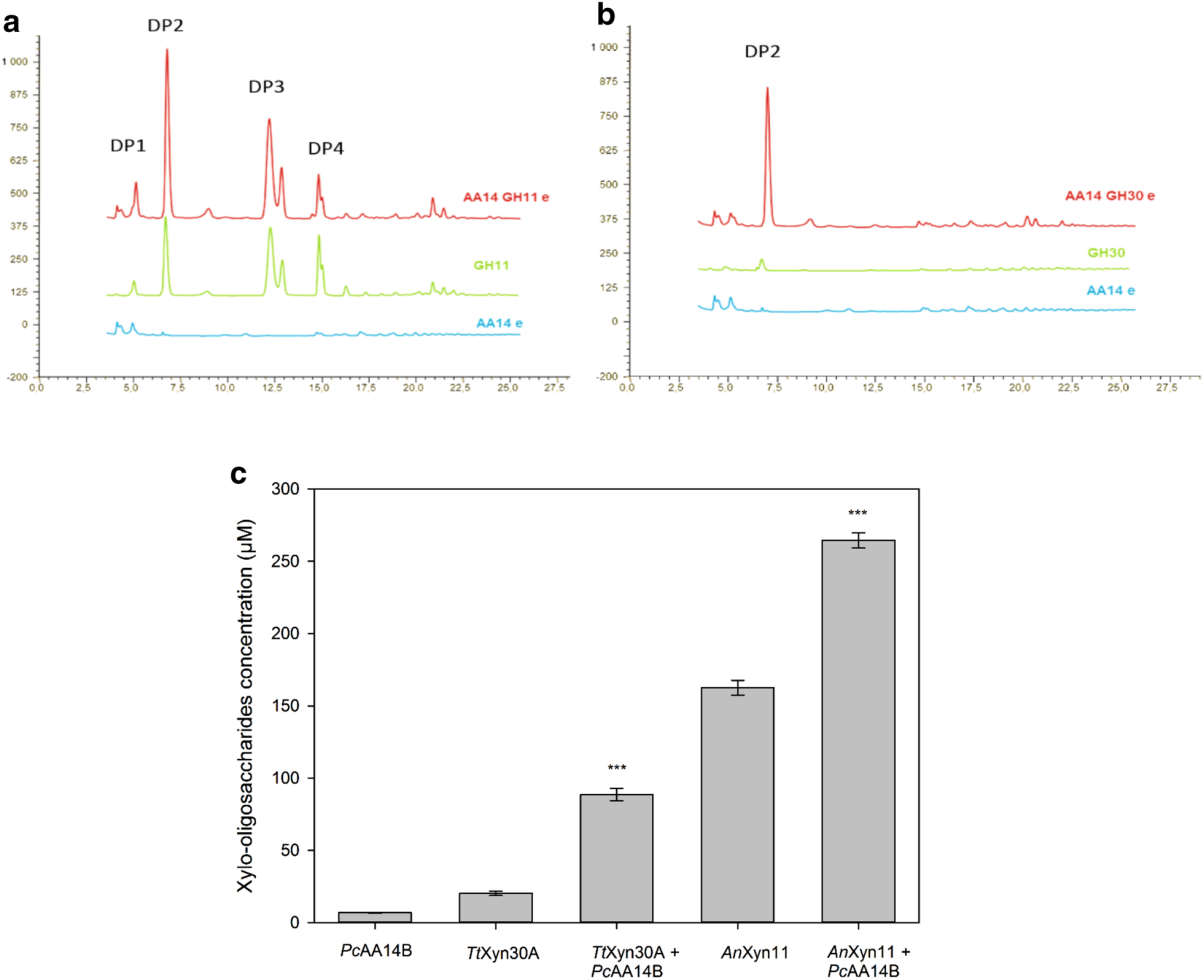
HPAEC analysis of released XOS from cellulosic fibers, after incubation with **a** *Pc*AA14A and *An*Xyn11 and **b** *Pc*AA14A and *Tt*Xyn30A. *Blue line*: *Pc*AA14A, *green line*: xylanase *An*Xyn11 or *Tt*Xyn30A, *red line*: *Pc*AA14A together with xylanase *An*Xyn11 or *Tt*Xyn30A. **c** Xylo-oligosaccharides release from cellulosic fibers after 16-h incubation with each enzyme combination. Error bars represent the standard deviation from three independent experiments. Asterisks (***p < 0.001) indicate significance based on Student’s *t* test

As previously reported, *Pc*AA14B LPMO acts synergistically with a family GH11 endoxylanase (*An*Xyn11) in the degradation of xylan-containing substrates [4]. In the present work, the synergism between *Pc*AA14B and *Tt*Xyn30A was first studied on wood cellulosic fibers, containing 20% xylan, similarly to Couturier et al. [4]. The cellulosic fibers’ substrate and the *An*Xyn11 xylanase were used as previously [4], to obtain comparable results. As shown in Fig. 1, the increase in XOS release was much higher in the case of *Tt*Xyn30A, compared to the synergistic action of *An*Xyn11 xylanase. More specifically, the increase in XOS release in the case of *Tt*Xyn30A was 338.6 ± 13.7%, corresponding to a degree of synergism (DS) 3.83 ± 0.08, while in the case of *An*Xyn11, the increase was only 62.8 ± 2.4%, corresponding to a DS of 1.60 ± 0.02. However, the release of total oligosaccharides was much higher with *An*Xyn11 (up to 264.5 μM, Additional file 1. Table S1) than with *Tt*Xyn30A (up to 88.6 μM, Additional file 1. Table S1), since the only xylooligosaccharide detected in the supernatant of *Tt*Xyn30A reactions was xylobiose.

**Fig. 2:**
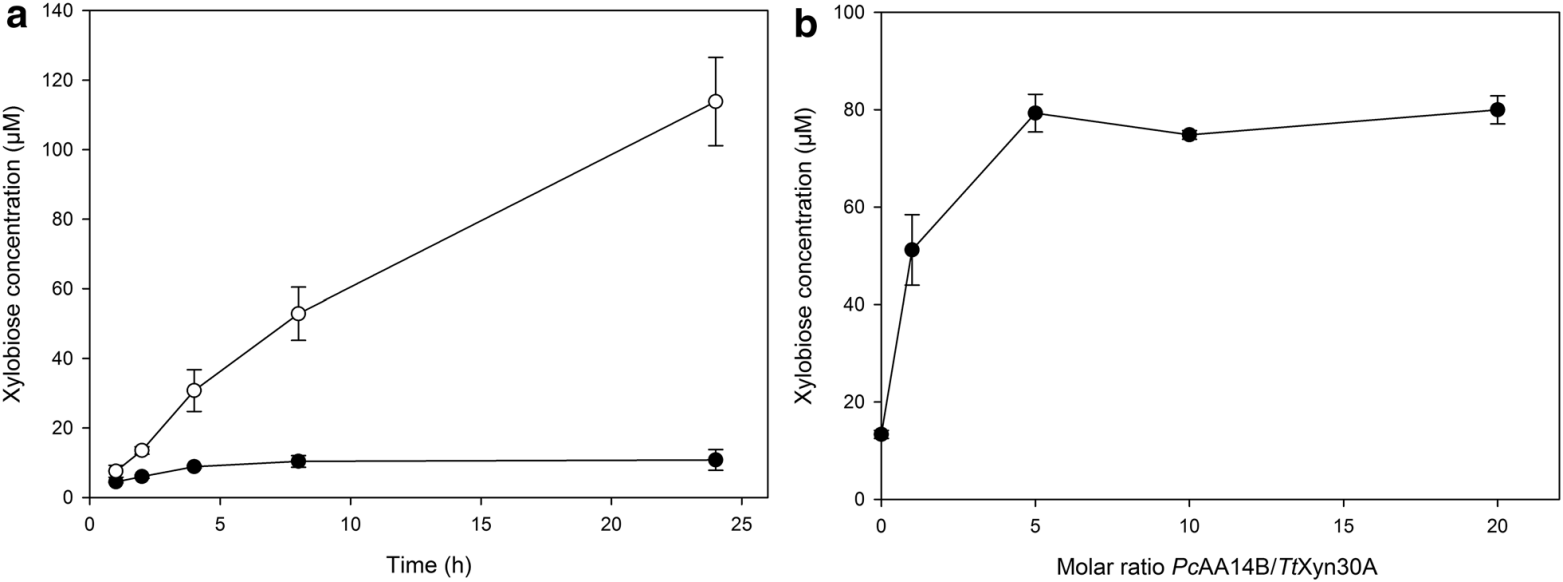
**a** Time course of the xylobiose release by *Tt*Xyn30A (filled circles) and *Tt*Xyn30A with *Pc*AA14B (open circles). **b** The effect of molar ratio of *Pc*AA14B to *Tt*Xyn30A on xylobiose release. *Tt*Xyn30A was added at a final concentration of 0.1 μM. Error bars represent the standard deviation from three independent experiments

To establish that the observed increase was indeed the result of a true synergistic relationship between *Pc*AA14B and *Tt*Xyn30A, the next step was to investigate the release of soluble XOS over time. Of note, as the major product of *Tt*Xyn30A is xylobiose, all quantifications were based on xylobiose concentration. As shown in Fig. 2a, the release of xylobiose reaches a plateau already after 4 h of reaction with the use of *Tt*Xyn30A alone, while with the addition of *Pc*AA14B the release of xylobiose is constantly increasing up to 24 h. This result is a strong indication that the action of *Pc*AA14B is constantly creating new initiation sites for *Tt*Xyn30A. From the above results, it is strongly suggested that *Tt*Xyn30A alone is not capable of hydrolyzing the recalcitrant part of xylan.

In an attempt to gain further insight on the synergistic relationship between these two enzymes, we tested the effect of *Pc*AA14B loading on the xylobiose release from *Tt*Xyn30A on cellulosic fibers. As shown in Fig. 2b, the addition of equimolar amount of *Pc*AA14B to *Tt*Xyn30A increased xylobiose release almost threefold, while increasing the molar ratio more than fivefold does not seem to increase further the synergy.

### Synergistic effects of *Pc*AA14B LPMO and *Tt*Xyn30A xylanase on lignocellulosic substrates

The synergism between *Pc*AA14Β LPMO and *Tt*Xyn30A xylanase was tested on different pretreated beechwood substrates. The substrates used in this study were selected based on their hemicellulose content, to gain better insights of the synergy of *Pc*AA14B and *Tt*Xyn30A in the degradation of cellulose-associated xylan. Milox process (substrates 3, 5 and 7) leads to the delignification of the material, with the concomitant production of lignin degradation products [19]. On the other hand, acetone/water oxidation (substrates 1, 2, 4 and 6) is a recently developed methodology, offering the advantage of higher delignification, while producing fewer degradation products [19]. Moreover, the parameters, including acetone to water ratio, can be optimized for the removal of hemicelluloses and lignin. For example, higher acetone content led to substrates with higher hemicellulose content (substrates 4 and 6), in contrast with high water content, leading to substrates (1 and 2), where most of the hemicellulose is removed. Overall, the selected substrates were considered representative of a wide variety of compositions in terms of cellulose, hemicelluloses, and lignin content. The properties of the substrates and the pretreatment conditions are shown in Table 1, together with the release of xylobiose and the degree of synergism. Examples of the analysis of soluble XOS released from lignocellulosic substrates, after treatment with *Pc*AA14B and *Tt*Xyn30A, are shown in Additional file 1: Fig. S1. During lignocellulosic substrates hydrolysis, xylobiose was the only product detected from the action of *Tt*Xyn30A, with HPAEC-PAD analysis. Therefore, all quantifications were made based on the exo-action of *Tt*Xyn30A, since uronic XOS were not detected in any case.

**Table 1:** Results on the synergism between *Pc*AA14B and *Tt*Xyn30A on different lignocellulosic substrates

Substrate #	Pretreatment conditions	Cellulose (% w/w)	Acid-insoluble lignin (% w/w)	Acid-soluble lignin (% w/w)	Hemicellulose (% w/w)	*Tt*Xyn30A (µM Xylobiose)	*Pc*AA14B + *Tt*Xyn30A (µM xylobiose)	Degree of synergism
1	d. H_2_O/acetone (50/50%), Air (40% O_2_), 8.5 bar (175 °C, 2 h)	79.47	8.4	0.5	4.01	1.23 ± 0.00	7.00 ± 0.30 (***)	5.70 ± 0.20
2	d. H_2_O/acetone (75/25%), Air (40% O_2_), 40 bar, 175 °C	84.10	11.5	1.0	5.18	0.98 ± 0.06	2.30 ± 0.07 (***)	2.35 ± 0.07
3	Milox 80 °C, 1 h, formic acid	80.30	6.3	1.0	12.35	26.7 ± 1.80	32.3 ± 1.3 (*)	1.21 ± 0.03
4	d. H_2_O/acetone (25/75%), Air (40% O_2_), 40 bar, 175 °C	75.36	2.1	1.6	17.93	36.1 ± 1.20	46.1 ± 2.3 (**)	1.28 ± 0.10
5	Milox 80 °C, 1 h, formic acid, 1 bar	75.31	2.1	1.2	19.21	13.9 ± 1.10	17.2 ± 1.1 (*)	1.24 ± 0.04
6	d. H_2_O/acetone (25/75%), Air, 20 bar	50.22	15.7	3.0	19.71	3.10 ± 0.25	2.20 ± 0.00 (*)	–
7	Milox 80 °C, 3 h, acetic acid	51.84	4.6	3.0	29.06	20.1 ± 1.00	18.0 ± 0.8 (ns)	–

The data on the pretreatment conditions and compositional analyses have been transferred by [19]. Error bars represent the standard deviation from three independent experiments. Asterisks (*p < 0.1; **p < 0.01; ***p < 0.001) indicate significance based on Student’s *t* test.

*ns* not significant

As shown in Table 1 and Additional file 1: Fig. S1, the increase in xylobiose release as a result of the synergism between *Pc*AA14B and *Tt*Xyn30A varies a lot among the different substrates. The highest release of xylobiose by *Tt*Xyn30A was observed on a substrate with relatively high hemicellulose content (substrate 4, Table 1), but, at the same time, the enhancement of *Tt*Xyn30A activity by *Pc*AA14B was low in this case. On the contrary, the lowest xylobiose release was observed in the substrates 1, 2 and 6. The low hemicellulose content might explain the low concentration of released xylobiose from *Tt*Xyn30A on substrates 1 and 2. Despite the low activity of *Tt*Xyn30A on both substrates 1 and 2, the relative increase in xylobiose release was the highest observed (Table 1). Therefore, it seems that the hemicellulose remaining after pretreatment is the most recalcitrant to degradation, since the activity of *Tt*Xyn30A on this substrate is the lowest observed. On the other hand, the high increase in xylobiose release could be the result of a high LPMO activity, and therefore, this recalcitrant part of hemicellulose seems to be an ideal substrate for *Pc*AA14B.

The synergistic activity of *Pc*AA14B and *Tt*Xyn30A seems to be interrupted in the case of substrates 6 and 7. Those are the substrates with the highest hemicellulose content tested in this study. The apparent loss of *Pc*AA14B activity on these substrates is a further indication of the substrate preference of the enzyme; high content of residual hemicellulose indicates that possibly the recalcitrant part of xylan, which is the assumed preferred substrate for *Pc*AA14B, is not accessible by the enzyme. Substrate 6 contains much more residual lignin, which may inhibit the performance of *Tt*Xyn30A due to its adsorption. To test this hypothesis, we performed a laccase treatment in substrate 6, aiming at the removal of residual lignin. The results showed that laccase treatment of substrate 6 increased xylobiose release from *Tt*Xyn30A by 19.7 ± 2.9%, indicating that residual lignin is responsible, at least to a certain extent, for *Tt*Xyn30A inhibition.

Products of the endoxylanase activity of *Tt*Xyn30A, such as 4-*O*-MeGlcA decorated XOS, were not detected in the above conditions. This could not only be attributed to their low concentration in the experimental setup of the present study, or to their low abundance in the beechwood substrates, but it could also indicate that the endo-action of *Tt*Xyn30A is inhibited by the structure of the recalcitrant twofold xylan that is bound with the underlying cellulose fibrils. In this case, the role of *Pc*AA14B could be much more crucial. To elucidate this, hydrolysis of substrates 1 and 4 was performed with increased concentration of *Tt*Xyn30A, in the absence of *Pc*AA14B. Positive controls with soluble beechwood xylan were also included. The samples were properly diluted and analyzed with HPAEC-PAD, and the chromatograms are shown in Additional file 1: Fig. S2. In the samples of substrate 1, no UXOS were detected, which can be explained by the low hemicellulose content of this substrate. On the other hand, in soluble beechwood substrate, UXOS were detected, and one of them was identified as 2,3-(4-*O*-methyl-α-D-glucuronyl)-xylotriose, against a suitable standard. In samples of substrate 4, one small peak appeared, with retention time corresponding to a possible UXOS product. The same peak was also detected in soluble beechwood xylan samples (Additional file 1: Fig. S2). This result corroborates with the higher hemicellulose content of substrate 4 and the higher activity of *Tt*Xyn30A, compared with the other tested substrates.

### Effect of sequential or simultaneous addition of enzymes on the hydrolytic reactions

**Fig. 3:**
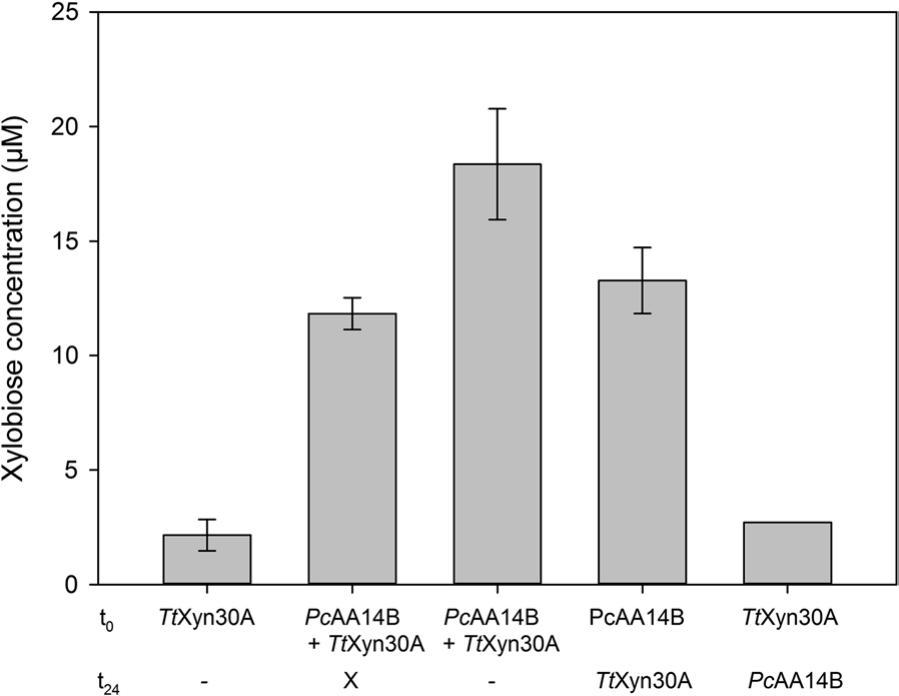
Time dependence of the synergism between *Pc*AA14A and *Tt*Xyn30A. The enzymes and their combinations were added at the specified time points, after boiling the reaction mixture to deactivate the first enzyme. The reaction was performed with substrate 1, and it was stopped at 48 h, except X, where the reaction was stopped at 24 h. Error bars represent the standard deviation from three independent experiments

Synergistic relationships among lignocellulose-acting enzymes are often time-dependent, and the order of enzyme addition can offer valuable insights regarding their mode of action on their polymer substrate. The effect of sequential or simultaneous addition of *Pc*AA14B and *Tt*Xyn30A was examined, and the results are shown in Fig. 3. The maximum release of xylobiose was shown when both enzymes were added simultaneously. In this case, the DS was found 1.96 ± 0.30 for the first 24 h, and 2.70 ± 0.09 when the reaction was prolonged for 48 h. When *Pc*AA14B was added first for 24 h prior to the addition of *Tt*Xyn30A, the DS was 1.93 ± 0.02, similarly to the case where both enzymes were added together and kept for 24 h. When *Tt*Xyn30A was added first for 24 h prior to the addition of *Pc*AA14B, no synergism was observed, and the xylobiose release reached 2.70 ± 0.00 μM (Fig. 3). This result is expected, since *Pc*AA14B does not release soluble products in detectable quantities. Therefore, an additional experiment was performed, where the substrate was pretreated with *Tt*Xyn30A and after 24 h, *Pc*AA14B was added, without deactivating the xylanase. The results were surprising, since the concentration of xylobiose release (2.80 ± 0.24 μM) was similar to the reaction when the xylanase was deactivated before LPMO addition. To determine the effect of enzyme stability to the above results, stability tests were performed for both enzymes under the same conditions. Both enzymes were found to be stable at 4 °C for 48 h, while *Tt*Xyn30A was also found stable at 45 °C during 48 h in the absence of substrate. *Pc*AA14b was found to maintain its activity fully for the first 24 h, but up to 48 h the residual activity drops to 55.9 ± 1.4%. The stability of *Tt*Xyn30A was also tested in the presence of substrate 1. Surprisingly, after 24 h at 45 °C, the residual activity of *Tt*Xyn30A dropped to 9.00 ± 0.57% of the initial, while after 48 h only traces of the initial activity were detected in the supernatant. This result could be due to unproductive binding of *Tt*Xyn30A to the substrate and specifically to the lignin part, which in substrate 1 amounts to 8.9% (w/w). This finding is in accordance with our results regarding the diminished activity of *Tt*Xyn30A in substrate 6 due to its high lignin content. Therefore, when *Tt*Xyn30A is added first, it is possible that the enzyme is already “deactivated” due to unproductive binding on lignin prior to *Pc*AA14B addition.

### Synergistic effects of *Pc*AA14B and cellobiohydrolases on the hydrolysis of lignocellulosic substrates

**Fig. 4:**
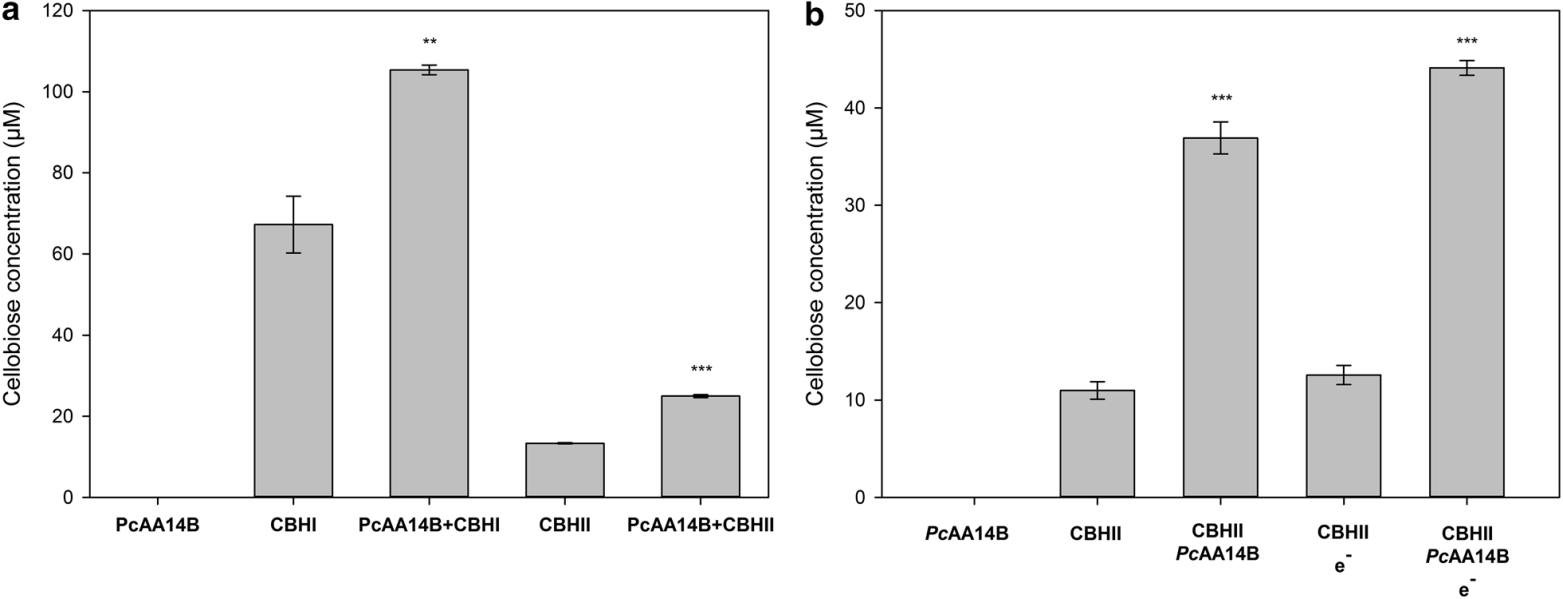
Synergism between *Pc*AA14B and cellobiohydrolases. **a** Synergistic effect of *Pc*AA14B supplementation on CBH-mediated hydrolysis of substrate 1. **b** Synergistic effect of *Pc*AA14B supplementation, with and without electron donor, on CBHII-mediated hydrolysis of microcrystalline cellulose (Avicel). Error bars represent the standard deviation from three independent experiments. Asterisks (**p < 0.01; ***p < 0.001) indicate significance based on Student’s *t* test

The next step was to explore the synergistic relationships of *Pc*AA14B with single cellulose-acting enzymes, since in our previous study, the increase of released glucose by a complex (hemi)cellulolytic cocktail by the addition of *Pc*AA14B was documented [4]. The synergism between *Pc*AA14B and cellobiohydrolases CBHI and CBHII was tested on substrate 1 (Table 1), since maximum *Pc*AA14B activity was observed using this substrate, in the previous experiments with *Tt*Xyn30A. The quantification of the results was based on cellobiose release, and the results are shown in Fig. 4a. *Pc*AA14B, although it is not active on cellulose, was shown to greatly increase the release of cellobiose by CBH from substrate 1, even though peaks corresponding to oxidized sugars, or xylo-oligomers, were not detected. Although the release of cellobiose was more prominent in the reaction of *Pc*AA14B with CBHI, the DS was slightly higher in combination with CBHII (1.58 ± 0.15 and 1.87 ± 0.01, respectively). The synergism between *Pc*AA14B and CBHII was further tested on microcrystalline cellulose (Avicel), with or without cysteine as electron donor, and the results are shown in Fig. 4b. The addition of *Pc*AA14B enhanced the production of cellobiose from CBHII, with or without the addition of electron donor, corresponding to DS of 3.70 ± 0.35 and 3.35 ± 0.40, respectively.

## Discussion

Regarding the enzymatic degradation of lignocellulosic materials, the ‘blocking effect’ of xylan on the accessibility of the underlying cellulose to cellulases is widely established in literature [20]. This is further supported by the number of studies on the synergistic effects of xylanases on cellulose hydrolysis by cellulases [15, 16, 21, 22]. However, a large part of xylan is still recalcitrant to degradation by most known enzymes, leading research not only toward the discovery of new enzymatic activities, but also on the exploration of new synergistic interactions among known enzymes [20]. In the present work, not only the synergistic interactions between the xylan-active LPMO *Pc*AA14B and the glucuronoxylan-specific xylobiohydrolase *Tt*Xyn30A, but also cellobiohydrolases, fill a significant knowledge gap on our understanding of the microbial degradation of (hemi)cellulose.

**Fig. 5:**
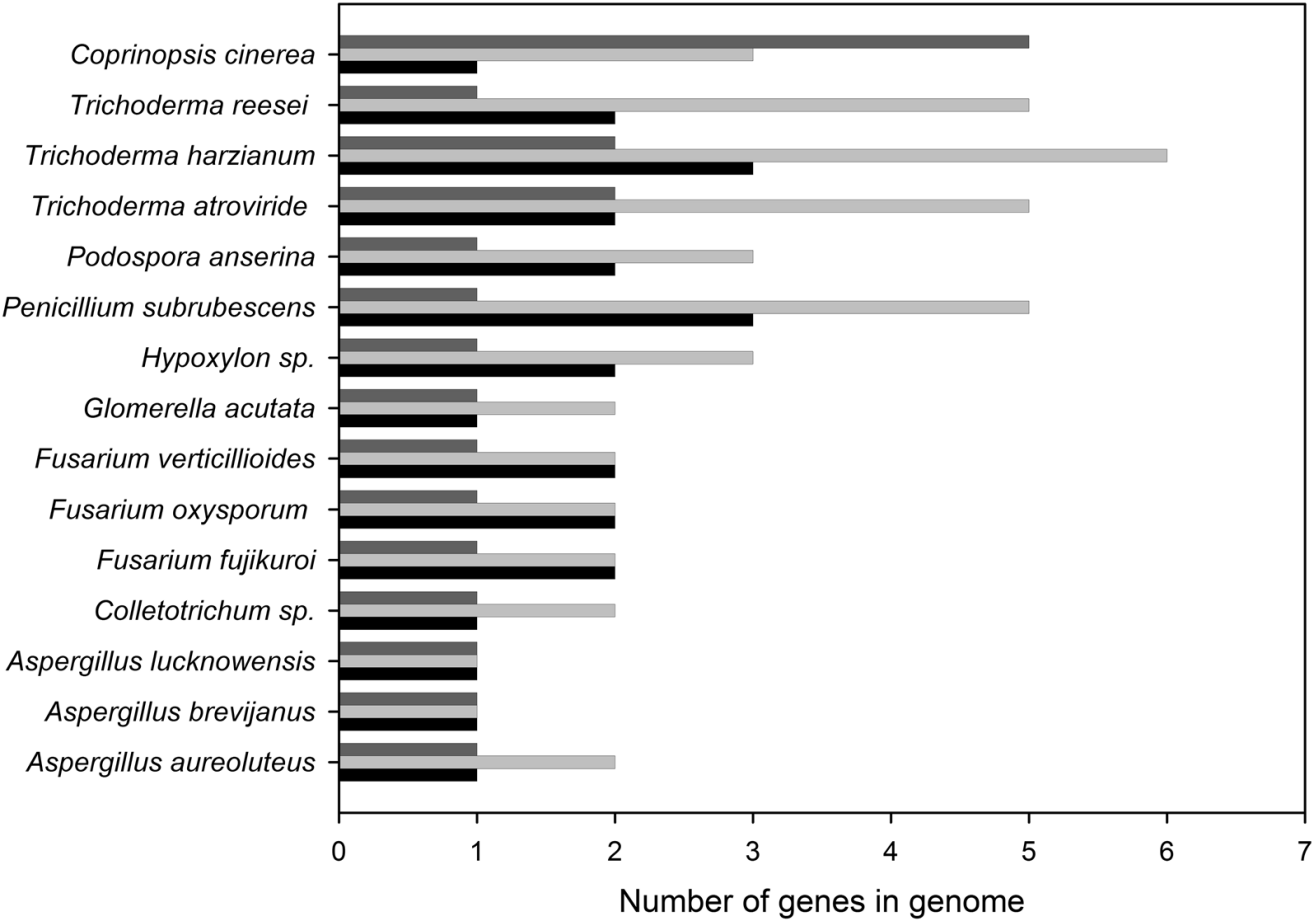
Number of genes belonging to either GH30 (light grey bars), GH30_7 (black bars) or AA14 (dark grey bars) CAZy (sub)family, in fungal genomes

Both AA14 and GH30_7 are enzyme classes found in fungal saprotrophs [2, 4]. Bioinformatics analyses among filamentous fungi revealed that AA14 and GH30_7 genes can both be found at the genomic level in some well-known fungal saprotrophs like *Trichoderma reesei, Podospora anserina* and *Fusarium oxysporum* (Fig. 5), suggesting that such synergistic activities could occur in nature.

**Fig. 6:**
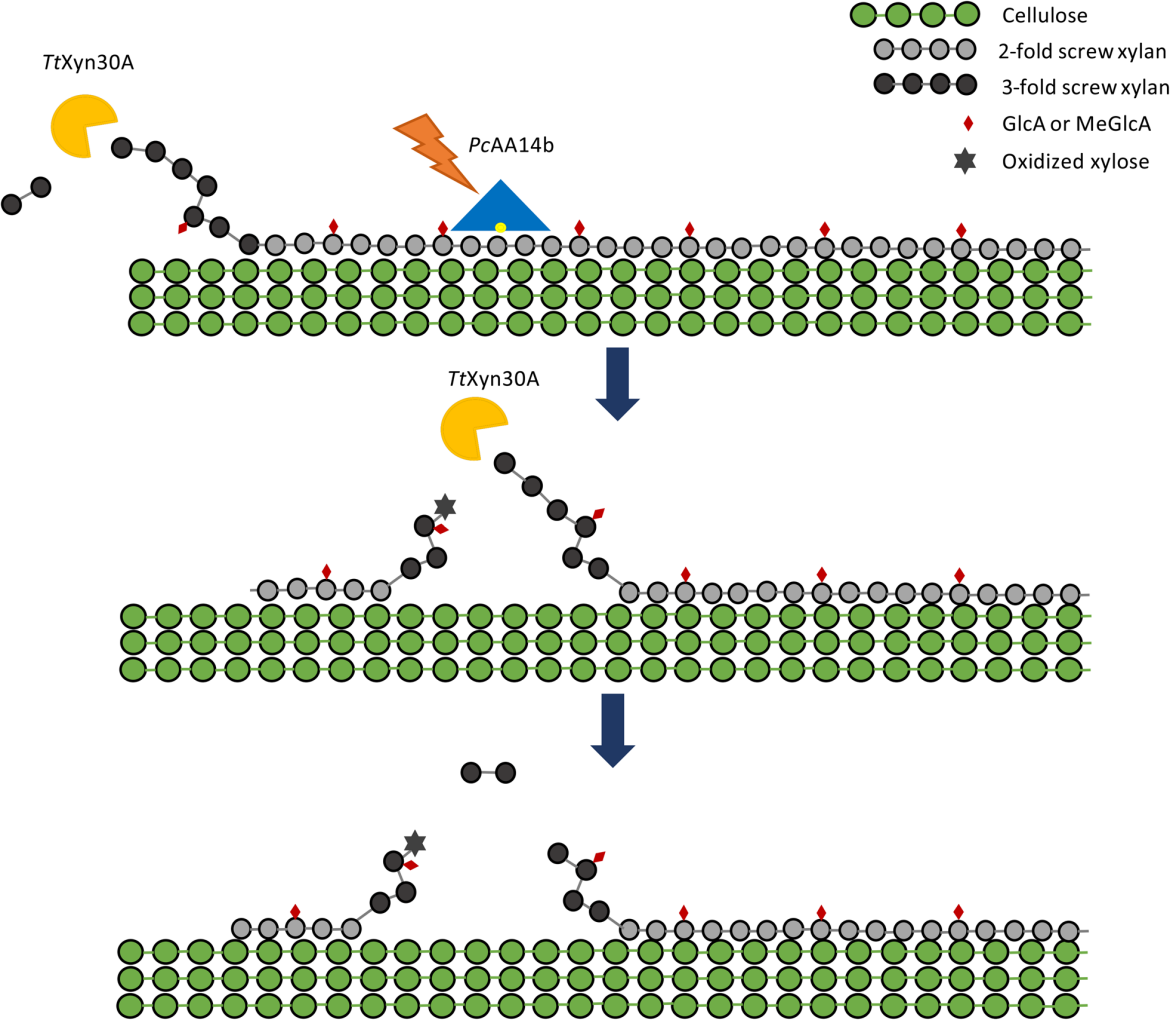
Schematic representation of the proposed hypothesis for the *Tt*Xyn30A–*Pc*AA14B synergistic action on xylan. The action of *Pc*AA14B on twofold recalcitrant xylan (*grey*) creates new free, threefold xylan ends (*black*) for the action of *Tt*Xyn30A. Τhe products of the endoxylanase activity of *Tt*Xyn30A (UXOS) are not shown, since they were not detected in this study

The synergistic effects observed between *Pc*AA14B and *Tt*Xyn30A on birchwood cellulosic fibers were significantly higher than those reported for *Pc*AA14B and a GH11 xylanase *An*Xyn11, as described by Couturier et al. [4] and repeated here for comparative purposes. The synergistic interaction between *Pc*AA14B and *Tt*Xyn30A was found to be dose-dependent, while the addition of *Pc*AA14B led to ongoing xylobiose release by *Tt*Xyn30A over a 24-h period, in contrast to the plateau reached by *Tt*Xyn30A only after 8 h, during hydrolysis of birchwood cellulosic fibers. Altogether these results led to the hypothesis that the action of *Pc*AA14B is constantly creating substrate sites available for *Tt*Xyn30A on the xylan polymer, as shown in Fig. 6.

The activity of *Tt*Xyn30A was tested in pretreated natural lignocellulosic substrates for the first time in this study. *Tt*Xyn30A also hydrolyzed the glucuronoxylan of most tested beechwood substrates with hemicellulose content >10% (w/w), releasing xylobiose. A single exception to this was observed during hydrolysis of substrate 6, which contained high levels of residual lignin. Residual lignin was found to inhibit *Tt*Xyn30A. Although cellulases inhibition by the presence of lignin, due to enzyme absorption on the surface of the polymer [23], is much more prominent, xylanase activity was also reported to be negatively affected [24, 25], probably due to ionic interactions between the charged moieties of the enzyme surface and lignin –COOH and –CO groups.

Regarding the activity of *Pc*AA14B, Couturier et al. proposed a specific hypothesis for the substrate preference of this LPMO [4]; *Pc*AA14B would be only active on the twofold screw xylan polymer that is attached to cellulose. Simmons et al. showed that the attachment of xylan polymers to underlying cellulose fibrils induces the twofold screw conformation on the xylan molecule, in contrast with the threefold screw conformation of soluble xylan [26]. Twofold screw xylan is a more rigid structure, resembling the cellulose fibrils, and therefore shows a certain degree of ‘crystallinity’ [6]. The results of our study strengthen the initial hypothesis, revealing a relationship between the hemicellulose content of the substrate, and the degree of synergism between the two enzymes; the lower the hemicellulose content, the higher the degree of synergism observed, at least for beechwood substrates, rich in glucuronoxylan. Moreover, the synergism among the two enzymes is less obvious in substrates with high hemicellulose content (e.g., 29% w/w), where the recalcitrant part of xylan is perhaps not accessible to *Pc*AA14B.

The high degree of synergism observed for cellulosic fibers and some of the tested beechwood substrates among *Pc*AA14B and *Tt*Xyn30A, especially when compared to the results with *Pc*AA14B and *An*Xyn11 xylanase, can also be justified by previous findings on the conformation of xylan in plant cell walls. Grantham et al. previously showed that xylan decorations, such as acetyl, arabinosyl and GlcA or 4-*O*-MeGlcA groups, are essential for the proper attachment of xylan to the underlying cellulose fibrils, and therefore, for the formation of the twofold xylan structure [6]. The oxidative cleavage of recalcitrant xylan by *Pc*AA14B created free ends, which in the case of glucuronoxylan are evenly decorated with 4-*O*-MeGlcA moieties. The newly created free ends function as substrate sites for the xylobiohydrolase activity of *Tt*Xyn30A, which ultimately releases xylobiose. The synergistic effect is further supported by the complementary action of both enzymes: while *Pc*AA14B oxidizes at the C1 position of the xylan backbone, *Tt*Xyn30A releases xylose dimers from the non-reducing end. This synergistic relationship is similar to the synergism observed between CBHs and cellulose-active LPMOs during cellulose hydrolysis [13]. Products of the endoxylanase activity of *Tt*Xyn30A, such as 4-*O*-MeGlcA decorated XOS, were detected in higher enzyme loadings, and in hemicellulose-rich substrates. In regard to the endoxylanase activity, *Tt*Xyn30A has been shown to attack the second glycosidic bond from the 4-*O*-MeGlcA position, while GH11 xylanases are well known to act several xylose monomers away from the substituent position, due to the architecture of the binding site cleft, which is not large enough to accommodate decorated xylans [27, 28]. This is also showed by Couturier et al., where the non-oxidized products found by the combined action of *Pc*AA14B and *An*Xyn11 were X_3_MeGlcA, X_4_MeGlcA and X_5_MeGlcA [4].

The synergism observed between *Tt*Xyn30A and *Pc*AA14B was found to be more prominent when both enzymes were added simultaneously rather than sequentially. This is not usual for enzymes acting on the same polymer, due to competition for binding sites. However, there are some reports showing a similar trend [29]. Simultaneous addition of enzymes with complementary activities usually is more favorable when the enzymes act on different polymers (e.g., cellulases and xylanases) [30]. Malgas et al. proposed an ‘alternating effect’ for the synergism displayed among cellulases and xylanases, where each enzyme hydrolyzes the cellulose and xylan moieties, covering each other in the complex biomass structure [31]. This seems to be the case for the synergism among *Tt*Xyn30A and *Pc*AA14B, as shown in Fig. 6. Time dependence experiments provide valuable insights regarding the mode of action of *Pc*AA14B and its synergism with *Tt*Xyn30A. When the substrate was pretreated with the LPMO and xylanase was added, after LPMO deactivation, for 24 h, the xylobiose release was the same as in the case where both enzymes worked together for 24 h. These data indicate that *Pc*AA14B action modifies the substrate of *Tt*Xyn30A, making it more susceptible to hydrolysis, similarly to cellulose-acting LPMOs and cellobiohydrolases [13]. According to our hypothesis, although both enzymes act on the same polymer, its conformation is not the same: *Pc*AA14B acts on the twofold helical screw xylan, creating free ends for *Tt*Xyn30A action, while *Tt*Xyn30A acts on the free non-reducing ends of the xylan polymer, due to its xylobiohydrolase activity. This synergistic relationship, aside from its obvious significance in the design of next-generation (hemi)cellulolytic cocktails, could also be employed for the production of oligosaccharides from residual biomass, which are shown to have significant prebiotic activity [32].

In the present work, the synergistic action of *Pc*AA14B and cellobiohydrolases CBHI and CBHII was demonstrated for the first time. As previously shown, *Pc*AA14B was found to enhance the glucose release from a (hemi)cellulolytic cocktail from *T. reesei*, but the synergism was not further studied with single enzymes [4]. Cellobiose release was found to be enhanced by the addition of *Pc*AA14B on substrate hydrolysis by CBHs, and this finding in is accordance with the previous results, where the disruption of wood fibers was observed by the action of *Pc*AA14B [4]. The authors hypothesized that the disruption of wood fibers revealed more substrate sites for cellulases, and the results of this work support this hypothesis. Moreover, the synergism between *Pc*AA14B and CBHII was shown for Avicel degradation. Τhis synergistic effect on Avicel can also be attributed to traces of recalcitrant xylan [33], the removal of which may facilitate cellulose hydrolysis by cellobiohydrolases, as proposed by Igarashi et al. [34].

## Conclusions

In the present study, new synergistic relationships were established: the xylan-active LPMO, represented in this study by *Pc*AA14B, and a novel GH30_7 glucuronoxylan-active xylanase with xylobiohydrolase activity, represented by *Tt*Xyn30A, were found to act synergistically on cellulosic substrates. Also, the activity of *Tt*Xyn30A was documented against pretreated lignocellulosic substrates for the first time. The addition of *Pc*AA14B in the reaction mixture together with *Tt*Xyn30A increased greatly the xylobiose release from pretreated lignocellulosic substrates. The synergistic effects were most prominent in substrates of relatively low hemicellulose content, indicating a preference of *Pc*AA14B for recalcitrant xylan. We hypothesized that the action of *Pc*AA14B on recalcitrant xylan creates new free ends in the polymer, which can be used as substrate for the action of *Tt*Xyn30A. *Pc*AA14B was also found to act synergistically with cellobiohydrolases CBHI and CBHII. Overall, the results of the present study provide additional insights into the mode of action of the *Pc*AA14B LPMO and will contribute to the design of next-generation enzymatic cocktails for the saccharification of woody lignocellulosic substrates with residual recalcitrant xylan.

## Methods

### Enzymes and substrates

Beechwood xylan, xylo-oligosaccharides (XOS), CBHI and CBHII cellobiohydrolases from *Trichoderma longibrachiatum* and a microbial source, respectively, and GH11 endo-1,4-β- xylanase M4 from *Aspergillus niger* (*An*Xyn11), were purchased from Megazyme (Bray, Co. Wicklow, Ireland). Kraft birchwood cellulosic fibers (79% (w/w) cellulose, 21% (w/w) xylan, [4]) were kindly provided by Sandra Tapin (FCBA, Grenoble, France). Aqueous dispersions of Kraft birchwood cellulosic fibers were adjusted to pH 5.2 with acetate buffer (50 mM) in a final reaction volume of 0.5 mL. Different beechwood lignocellulosic substrates were produced from commercially available beechwood (Lignocel® HBS 150–500), pretreated, as previously described [19]. *Tt*Xyn30A was produced and purified as described in [2]. *Pc*AA14B was produced and purified, as previously described [4]. In brief, *P. pastoris* clones bearing the recombinant proteins in frame with (His)_6_-tag sequences were grown overnight at 30 °C in BMGY medium. The cells were then transferred to BMMY medium supplemented with methanol, as described in EasySelect™ Pichia Expression kit, and incubated 3–6 days for enzyme production. Then, the supernatants were collected by centrifugation, and they were filtered, concentrated and dialyzed. Both enzymes were purified with immobilized metal ion affinity chromatography (IMAC), to homogeneity.

### Bioinformatics analyses

Bioinformatics analyses were performed on sequenced fungal genomes publically available (NCBI non-redundant sequence database and JGI). The co-occurrence of GH30_7 and AA14 modules in fungal genomes was searched using CAZy tools (www.cazy.org).

### Synergism assays

Cellulosic fibers, lignocellulosic substrates, or Avicel were added to 50 mM sodium acetate buffer, pH 5.2 at a final concentration of 0.5% (w/v). The enzymes were added at a final concentration of 2 μM for *Pc*AA14B and 0.1 μΜ for *Tt*Xyn30A, *An*Xyn11, CBHI or CBHII, unless stated otherwise. Cysteine was used as an electron donor on the assays using birchwood cellulosic fibers and Avicel, at a final concentration of 1 mM. The reactions (0.5 mL) were incubated for 16 h in 45 °C, at 850 rpm, in a thermomixer (Eppendorf, Montesson, France). The reaction was terminated with 10-min incubation in 100 °C, and the reactions were centrifuged to remove the insoluble fraction.

The hydrolysis products were analyzed with high-performance anion-exchange chromatography (HPAEC), using a CarboPac PA-1 (4 × 250 mm, Dionex) column with a pulsed amperometric detector (PAD) equipped with a gold electrode. The solvents used were NaOH 100 mM (solvent A) and NaOAc (1 M) in 100 mM NaOH (solvent B) at a flow rate of 1 mL min^−1^. For the analysis of oligosaccharides, the following gradient was applied: 0–10 min 0–10% B, 10–35 min 10–30% B, 35–40 min 30–100% B 40–41 min 100–0% B, 41–50 min isocratic step, 100% A. Xylobiose, XOS and cellobiose were quantified against suitable standard curves.

The degree of synergism was calculated according to the following equation:

$$\mathrm{DS}=\frac{{X}_{A,B}}{{X}_{A}+{X}_{B}}$$

where ${X}_{A,B}$ is the μM of product from the action of both enzymes on the substrate, ${X}_{A}$ the μM of product from the action of the LPMO and ${X}_{B}$ the μM of product from the action of xylanases *Tt*Xyn30A or *An*Xyn11, or cellobiohydrolases CBHI and CBHII.

The time dependence of the synergism between *Tt*Xyn30A and *Pc*AA14B was tested, as mentioned above. First, a pretreatment of 10 mg mL^−1^ of substrate 1 was performed with either *Tt*Xyn30A (0.1 μM) or *Pc*AA14B (2 μM) at 45 °C for 24 h. Then, the first enzyme was deactivated by boiling the reaction mixture, the second enzyme was added (*Pc*AA14B or *Tt*Xyn30A), and the reaction was incubated for a further 24 h. The effect of *Tt*Xyn30A alone and together with *Pc*AA14B added together from the start of the reaction and incubated for 24 or 48 h were used as controls. Xylobiose concentrations were determined with HPAEC-PAD, as mentioned above.

The synergism of *Pc*AA14B with cellobiohydrolases CBHI and CBHII was studied, as mentioned above. In brief, CBHI and CBHII were added at a final concentration of 0.1 μM, while *Pc*AA14B was added at a final concentration of 1 μM, to 15 mg mL^−1^ of substrate 1. Cysteine (1 mM) was added as electron donor, as indicated. The reaction was incubated at 45 °C for 16 h, and then the samples were boiled and filtered before HPAEC-PAD analysis. Cellobiose quantification was performed against a suitable calibration curve.

### Detection of UXOS in the reaction supernatants of *Tt*Xyn30A

For the detection of UXOS products of *Tt*Xyn30A, 50 mg mL^−1^ substrate 1 and 4 were treated with 0.25 mg mL^−1^ *Tt*Xyn30A for 24 h in 50 °C and 1100 rpm. Beechwood xylan (5 mg mL^−1^) was hydrolyzed with 0.02 mg mL^−1^ *Tt*Xyn30A in the same conditions. The reactions were terminated by boiling the samples. The samples from hydrolysis of substrates 1 and 4 were diluted 2 times before HPAEC-PAD analysis, while the samples from beechwood xylan hydrolysis were diluted 10 times before analysis. HPAEC-PAD analysis was performed with a different elution program [2]. The solvents used were NaOH 100 mM (solvent A) and NaOAc (0.5 M) in 100 mM NaOH (solvent B) at a flow rate of 1 mL min^−1^. For the analysis of oligosaccharides, the following gradient was applied: 0–5 min A, 5.1–30 min 0–20% B, 30.1–35 min 100% B, 35.1–50 min 100% A.

### Stability measurements

The stability of *Pc*AA14B and *Tt*Xyn30A was determined as follows: both enzymes were incubated in sodium acetate buffer, 50 mM, pH 5.2 for 48 h either in 4 or 45 °C. The final concentrations of enzymes were 2 μM for *Pc*AA14B and 0.1 μM for *Tt*Xyn30A. Samples were withdrawn in time points 0, 24 and 48 h, and the activity was determined. For *Tt*Xyn30A, the activity was measured with beechwood xylan as substrate, at a final concentration of 0.5% (w/v), for 24 h in 45 °C and 1200 rpm. The reducing sugars were quantified with the dinitrosalicylic acid method (DNS, [35]). In addition, the stability of *Tt*Xyn30A was measured also in the presence of 10 mg mL^−1^ substrate 1.

For *Pc*AA14B, due to the lack of soluble products, the activity was determined by measuring the degree of synergism with *Tt*Xyn30A. In the *Pc*AA14B-containing samples, substrate 1 was added at a final concentration of 10 mg mL^−1^ and fresh *Tt*Xyn30A at a final concentration of 0.1 μM. The mixture was incubated at 45 °C, and 1200 rpm for 24 h, and the xylobiose concentration was quantified with HPAEC-PAD, as described previously.

### Laccase treatment

A treatment with laccase enzyme was performed in substrate 6 (Table 1). Laccase Novozym^®^ 51003 was used at 10 U g^−1^ substrate, which was added at a final concentration of 10% (w/v). The mixture was incubated for 24 h in 40 °C, pH 6, 950 rpm, and then the supernatant was removed. The laccase-treated substrate was washed three times with ultrapure water, to remove low-MW phenolic compounds released by the action of the enzyme, and freeze-dried. Finally, it was hydrolyzed by *Tt*Xyn30A, as described previously. *Tt*Xyn30A was added at a final concentration of 1 μM.

### Statistical analysis

All experiments were performed in triplicate, and the results are expressed as mean value ± standard deviation. The statistical analysis was performed with SigmaPlot 12.5 software. Student’s *t* test was applied to experiment subsets, with the use of GraphPad Prism 5 software. *P* values less than 0.05 were considered statistically significant.

## Supplementary information

**Additional file 1: Table S1.** Xylo-oligosaccharide release from cellulosic fibers, after hydrolysis with *Pc*AA14B, *Tt*Xyn30A, *An*Xyn11 and their combinations, as shown in Fig. 1. Error bars represent the standard deviation from three independent experiments. **Figure S1.** Analysis of XOS release by *Pc*AA14B and *Tt*Xyn30A, in different pretreated lignocellulosic substrates, as shown in Table 1; (a) Substrate 5, (b) substrate 2, (c) substrate 4. Green line: substrate only, black line: *Pc*AA14B, red line: *Tt*Xyn30A, blue line: *Pc*AA14B and *Tt*Xyn30A. DP2: xylobiose. **Figure S2.** Analysis of XOS release by *Tt*Xyn30A, in beechwood xylan (10X diluted, blue line: reaction, light blue line: substrate blank) and substrate 4 (2X diluted, green line: reaction, yellow line: substrate blank); red line: 23-(4-O-Methyl-α-D-Glucuronyl)-xylotriose, black line: XOS standards (DP 1-5).
