# Supplementary material for: A new synergistic relationship between xylan-active LPMO and xylobiohydrolase to tackle recalcitrant xylan
Source: Biotechnol Biofuels. 2020 Aug 10;13:142. doi: 10.1186/s13068-020-01777-x (PMC7419196; doi:10.1186/s13068-020-01777-x)
Supplement: Supplementary file 1 — Additional file 1: Table S1. Xylo-oligosaccharide release from cellulosic fibers, after hydrolysis with PcAA14B, TtXyn30A, AnXyn11 and their combinations, as shown in Fig. 1. Error bars represent the standard deviation from three independent experiments. Figure S1. Analysis of XOS release by PcAA14B and TtXyn30A, in different pretreated lignocellulosic substrates, as shown in Table 1; (a) Substrate 5, (b) substrate 2, (c) substrate 4. Green line: substrate only, black line: PcAA14B, red line: TtXyn30A, blue line: PcAA14B and TtXyn30A. DP2: xylobiose. Figure S2. Analysis of XOS release by TtXyn30A, in beechwood xylan (10X diluted, blue line: reaction, light blue line: substrate blank) and substrate 4 (2X diluted, green line: reaction, yellow line: substrate blank); red line: 23-(4-O-Methyl-α-D-Glucuronyl)-xylotriose, black line: XOS standards (DP 1-5). [file 13068_2020_1777_MOESM1_ESM.pdf]

Supplementary material

A new synergistic relationship between a xylan-active LPMO and xylobiohydrolase to tackle recalcitrant xylan

Anastasia Zerva[1,2], Christina Pentari[1], Sacha Grisel[2], Jean-Guy Berrin[2], Evangelos Topakas[1*]

[1]*Industrial Biotechnology & Biocatalysis Group, School of Chemical Engineering, National Technical University of Athens, 9 Iroon Polytechniou Str., Zografou Campus, Athens 15780, Greece*

[2]*INRAE, Aix Marseille Univ., Biodiversité et Biotechnologie Fongiques (BBF), UMR1163, F-13009 Marseille, France*

*Correspondence to: E. Topakas. Tel: +30-210-7723264; fax: +30-210-7723163; e-mail: vtopakas@chemeng.ntua.gr and J-G. Berrin. Tel: +33491828604; fax: +33491828601: jean-guy.berrin@inra.fr

Table S1. Xylo-oligosaccharide release from cellulosic fibers, after hydrolysis with *Pc*AA14B, *Tt*Xyn30A, *An*Xyn11 and their combinations, as shown in Fig. 1. Error bars represent the standard deviation from three independent experiments.

Enzymes	***Xylo-oligosaccharide concentration (μM)***
***Pc*AA14B**	6.8 ± 0.3
***Tt*Xyn30A**	20.2 ± 1.6
***Tt*Xyn30A + *Pc*AA14B**	88.6 ± 4.3
***An*Xyn11**	162.5 ± 5.1
***An*Xyn11 + *Pc*AA14B**	264.5 ± 5.1

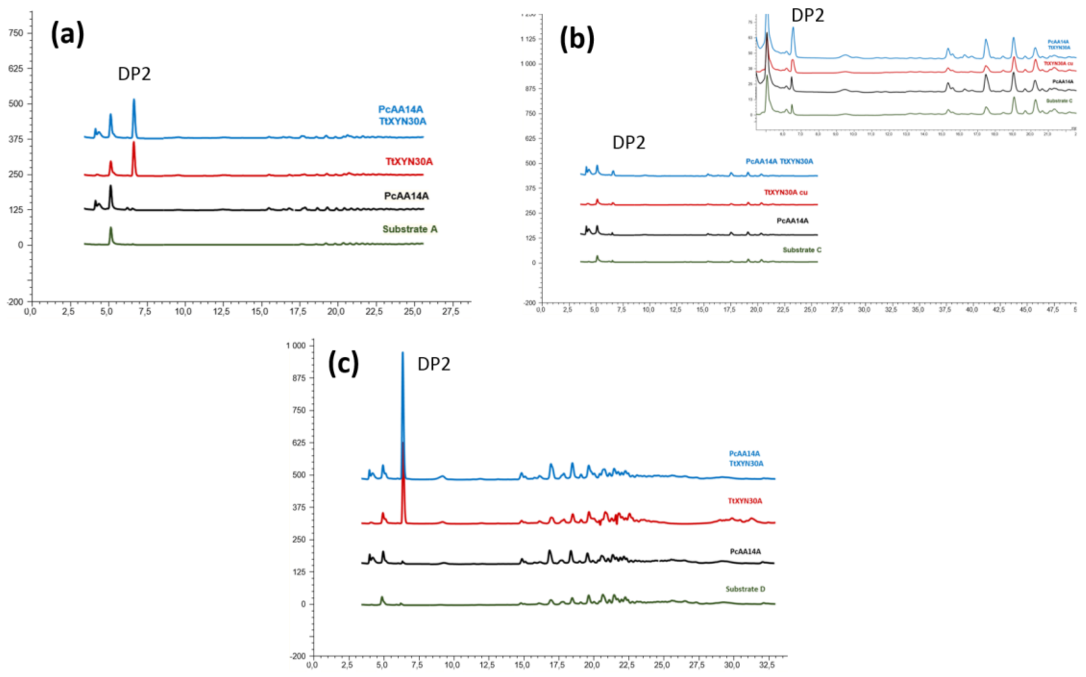

Fig. S1. Analysis of XOS release by *Pc*AA14B and *Tt*Xyn30A, in different pretreated lignocellulosic substrates, as shown in Table 1; (a) Substrate 5, (b) substrate 2, (c) substrate 4. *Green line*: substrate only, *black line*: *Pc*AA14B, *red line*: *Tt*Xyn30A, *blue line*: *Pc*AA14B and *Tt*Xyn30A. DP2: xylobiose.

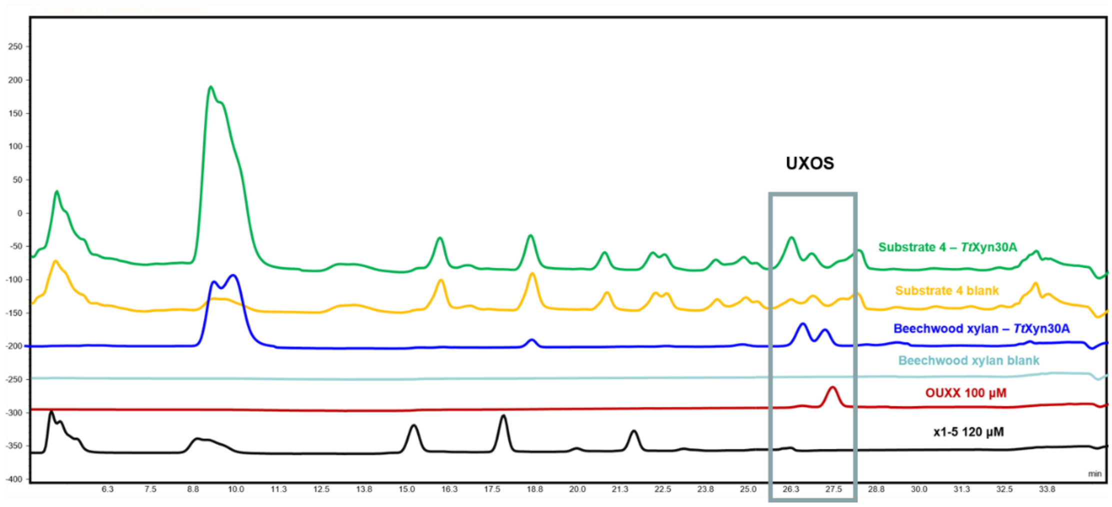

Fig. S2. Analysis of XOS release by *Tt*Xyn30A, in beechwood xylan (10X diluted, *blue line*: reaction, *light blue line*: substrate blank) and substrate 4 (2X diluted, *green line*: reaction, *yellow line*: substrate blank); *red line*: 23-(4-O-Methyl-α-D-Glucuronyl)-xylotriose, *black line*: XOS standards (DP 1-5).
